# Oglethorpe Medical College

**Published:** 1858-04

**Authors:** 


### Oglethorpe Medical College.

We learn from the Savannah Republican, that at the recent annual commencement of this Institution, the annual report represented "the School in a highly flourishing condition, with daily increasing prospects of permanency and usefulness."

The Degree of Doctor of Medicine was conferred upon eleven young gentlemen—six of whom were from Georgia; four from South Carolina; and one from Tennessee. The number of the class is not stated, but we learn from another source, that it was between thirty and forty.

